# Supplementary material for: Probing electrochemical reactions in organic cathode materials via in operando infrared spectroscopy
Source: Nat Commun. 2018 Feb 14;9:661. doi: 10.1038/s41467-018-03114-1 (PMC5812995; doi:10.1038/s41467-018-03114-1)
Supplement: Supplementary file 1 — Supplementary Information [file 41467_2018_3114_MOESM1_ESM.pdf]

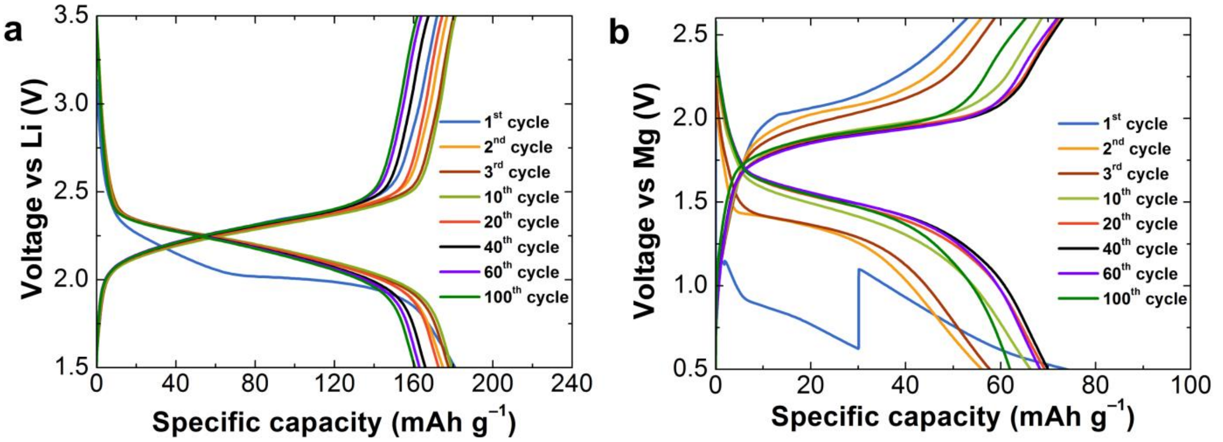

Supplementary Fig. 1 Galvanostatic discharge/charge cycles. **a** Galvanostatic discharge/charge cycles for the Li-PAQS and **b** for the Mg-PAQS system at 225 mA g^{-1} (1C) current density.

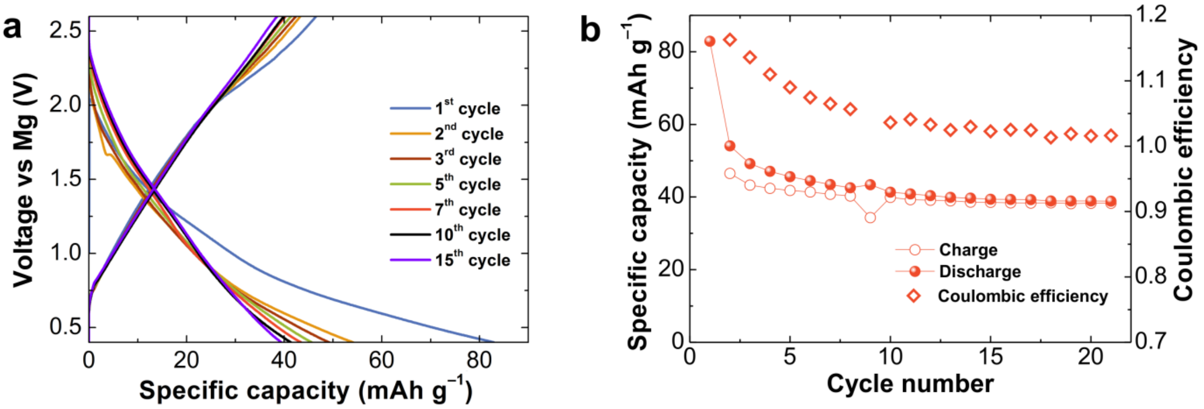

Supplementary Fig. 2 Electrochemical performance for Printex XE2 carbon black. The cathode is made out of Printex XE2 carbon black and PTFE binder in Mg system with 0.4 M $Mg(TFSI)_2$ 0.4 M $MgCl_2$ in TEG:DOL electrolyte at 11.3 mA g^{-1} (C/10) current density.

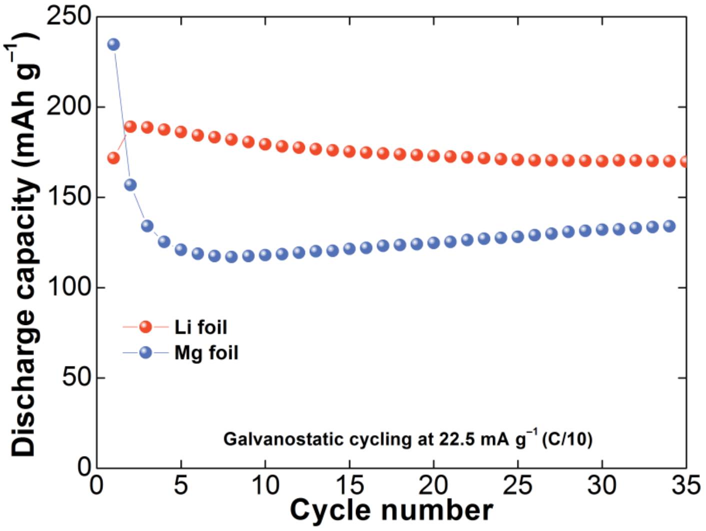

Supplementary Fig. 3 Discharge capacity for PAQS in Li (red) and Mg (blue) systems. Batteries were cycled at 22.5 mA g^{-1} (C/10) current density.

a b

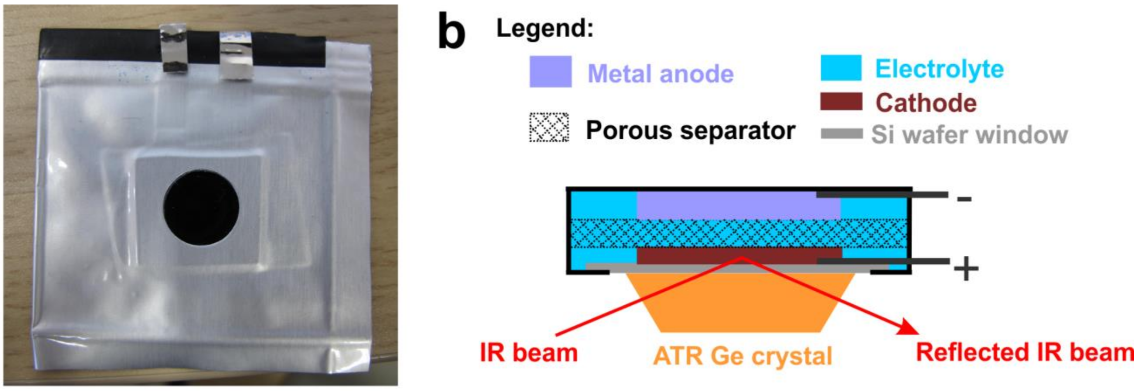

Supplementary Fig. 4 Design of the ATR-IR cell. **a** Photo picture of a two electrode spectroelectrochemical *in-operando* ATR-IR pouch cell with an IR-transmissive silicon wafer window and **b** the schematic cross-section view of the modified pouch cell on the ATR Ge crystal.

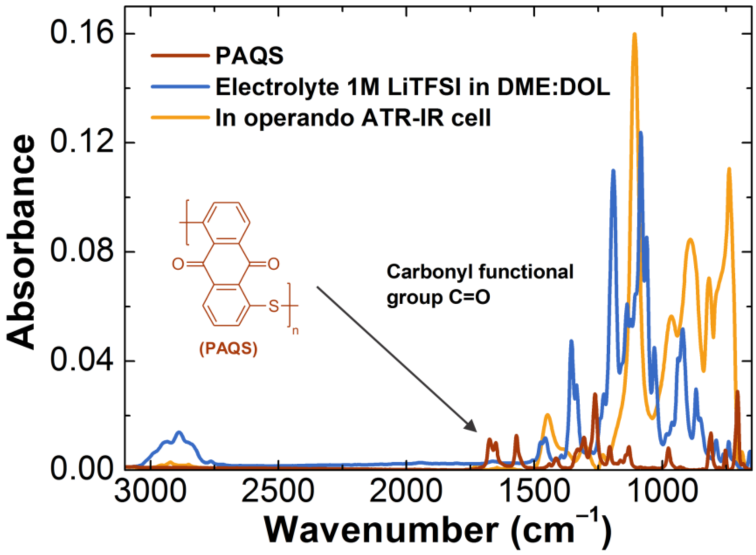

Supplementary Fig. 5 Comparison between the ATR-IR spectra of the organic material, electrolyte and cell. The PAQS is in brown, 1M LiTFSI in DME:DOL (vol.% 1:1) electrolyte is in blue and the *in-operando* ATR-IR cell is in orange.

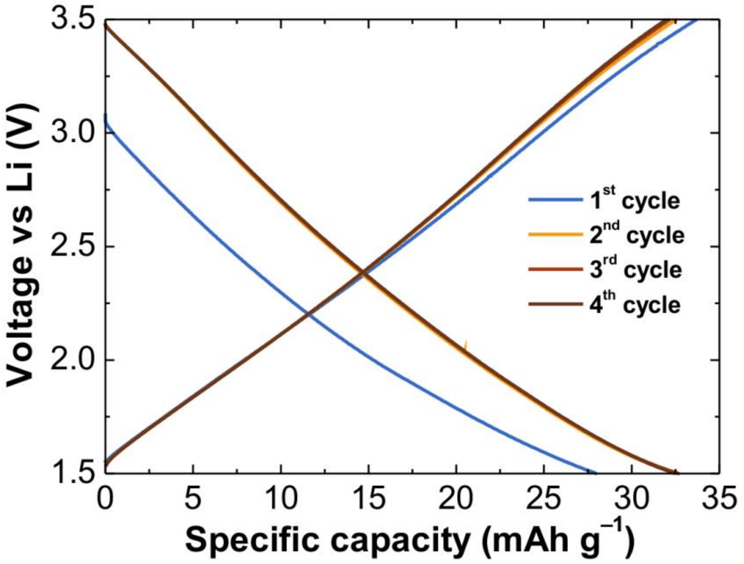

Supplementary Fig. 6 Galvanostatic discharge/charge for Printex XE2 in Li system. Galvanostatic discharge/charge curves for the 1st, 2nd, 3rd and 4th cycle for the cathode made out of Printex XE2 carbon black and PTFE binder (Printex XE2:PTFE = 70:30) with Li anode in the *in-operando* ATR-IR cell.

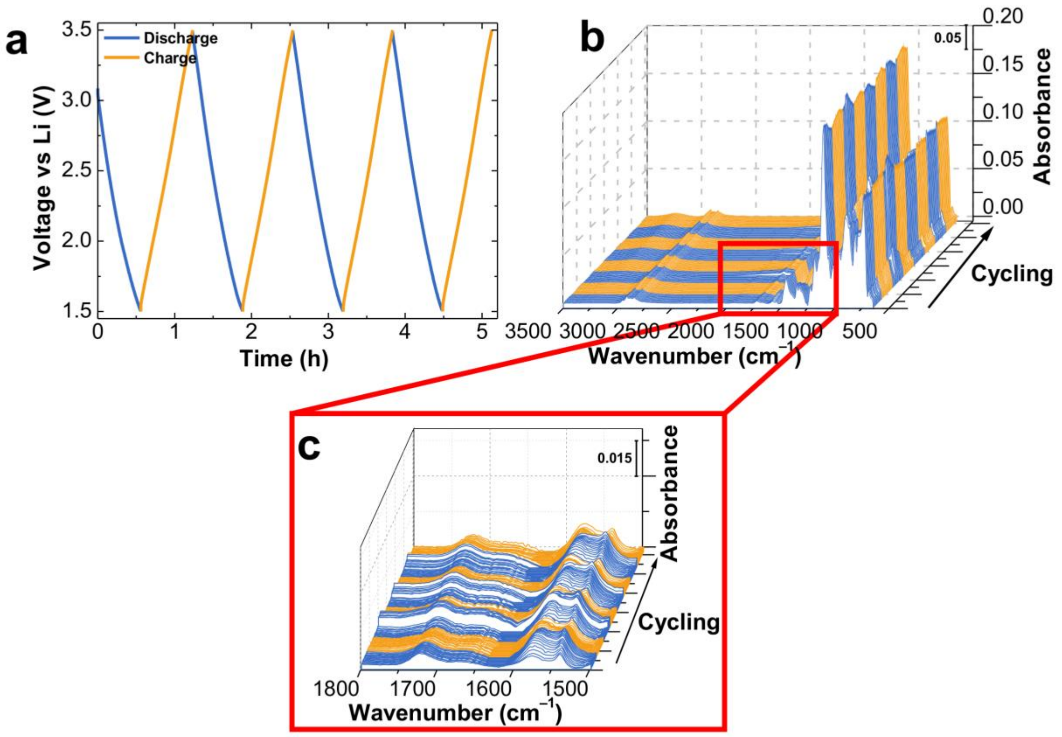

Supplementary Fig. 7 Electrochemical and IR characterization of Printex XE2 in Li system. **a** Voltage profile for Printex XE2:PTFE = 70:30 (discharge in blue, charge in orange); **b** corresponding ATR-IR spectra of Printex XE2:PTFE = 70:30 during galvanostatic cycling in a region from 3500 to 550 cm^{-1}; **c** magnification of the ATR-IR spectra in the region from 1800 to 1500 cm^{-1}.

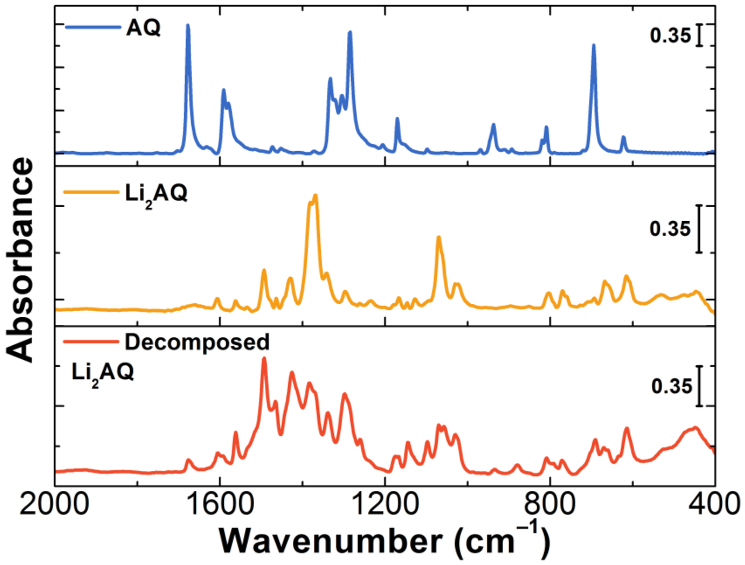

Supplementary Fig. 8 IR spectra for the AQ and synthesised Li_2AQ. IR spectra of AQ in KBr pellet (light blue), of Li_2AQ in nujol (orange) and IR spectra of decomposed Li_2AQ in nujol (red).

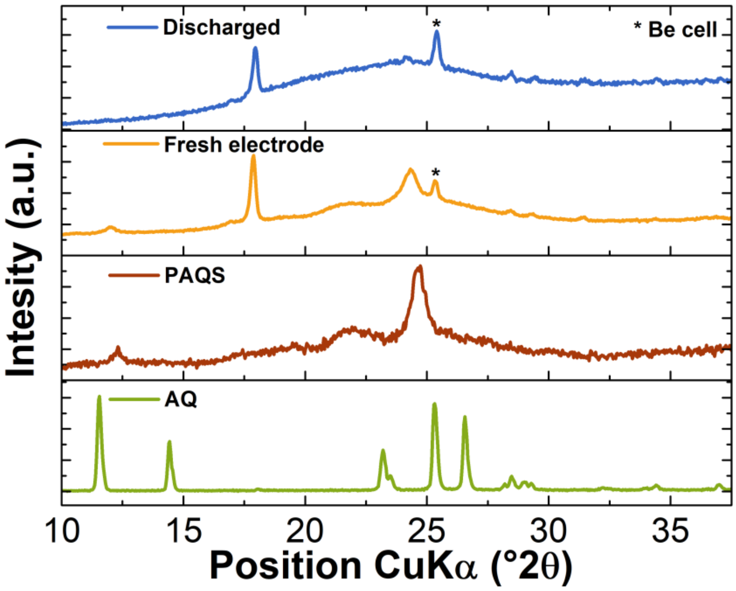

Supplementary Fig. 9 XRD measurements. AQ (green diffractogram), synthesised PAQS (red diffractogram), fresh PAQS cathode (orange diffractogram) and *ex-situ* PAQS cathode in a discharge state (blue diffractogram). The XRD of the fresh cathode and the *ex-situ* cathode in a discharge state were measured inside a Swagelok type cell with a beryllium window.

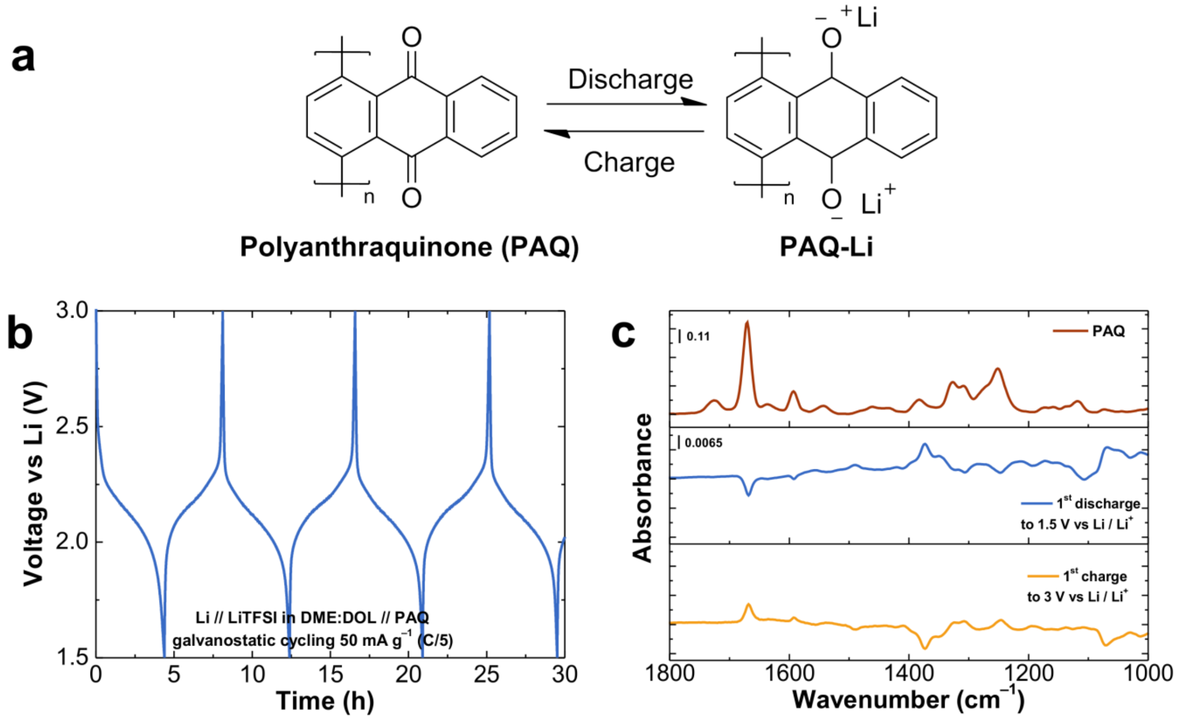

Supplementary Fig. 10 Electrochemical and IR characterization for PAQ in Li system.
a Proposed electrochemical mechanism for polyanthraquinone (PAQ) in lithium battery system; **b** galvanostatic cycling in voltage range from 1.5 to 3.5 V vs. Li/Li^+ in the *in-operando* ATR-IR in lithium battery cell; **c** ATR-IR spectrum of PAQ (brown), differential spectrum of first discharge (blue) and differential spectrum of first charge (yellow).

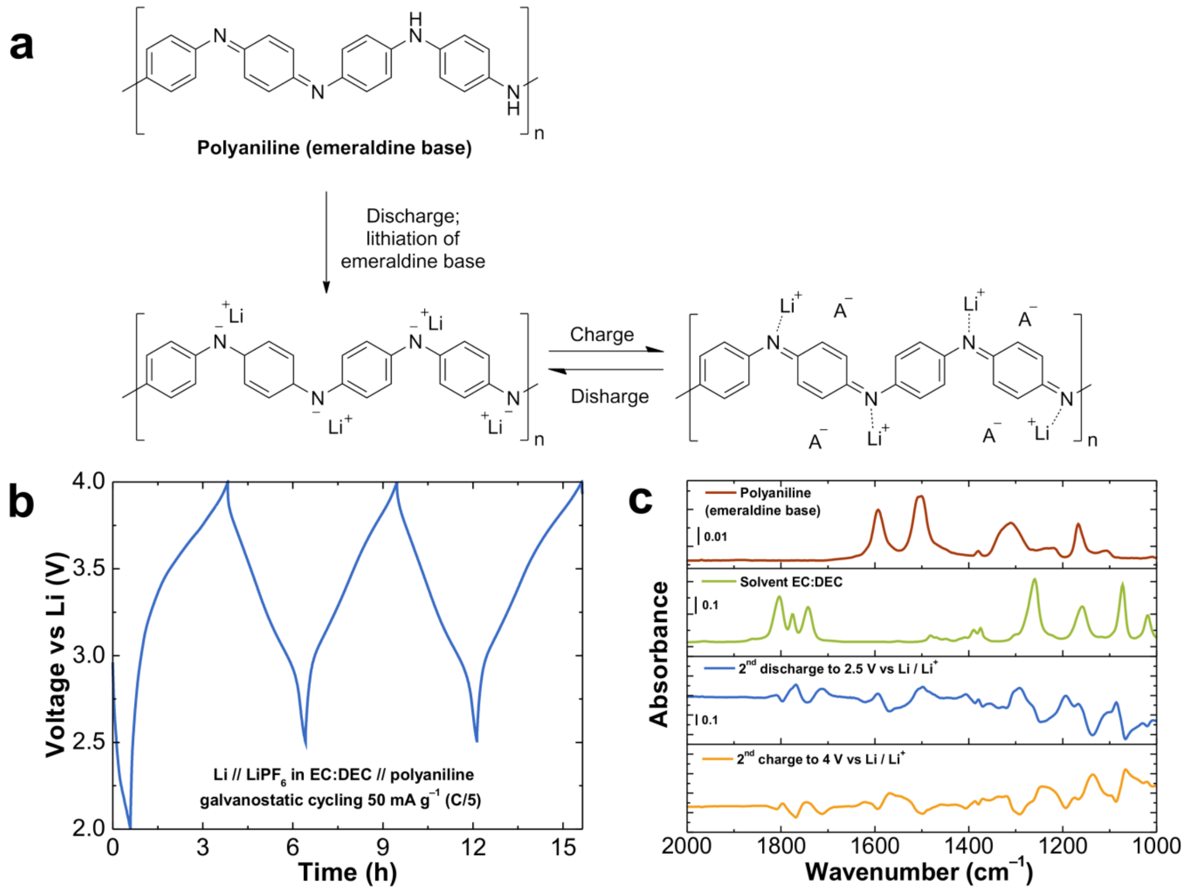

Supplementary Fig. 11 Electrochemical and IR characterization for polyaniline in Li system. **a** Polyaniline (emeraldine base) structure and proposed electrochemical reaction mechanism; **b** galvanostatic cycling in the voltage range from 2.5 (except first cycle to 2.0 V to achieve lithiation of emeraldine base) to 4.0 V Li/Li^+ in the *in-operando* ATR-IR in lithium battery cell; **c** ATR-IR spectrum of polyaniline-emeraldine base (brown), EC:DEC solvent (green), differential spectrum of second discharge (blue) and differential spectrum of second charge (yellow).

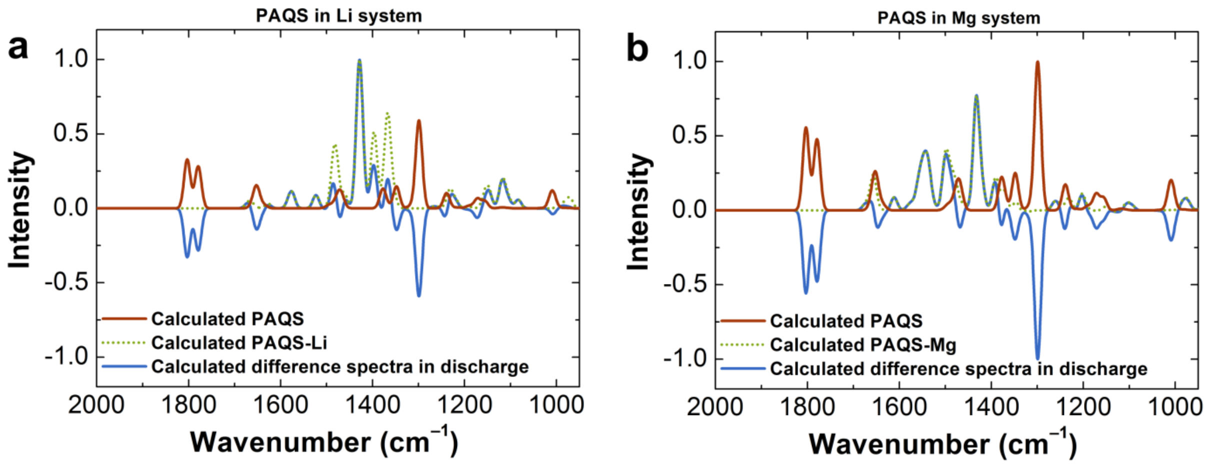

Supplementary Fig. 12 Theoretical spectra of PAQS and $PAQS^{2-}$ in Li and Mg system. The theoretical spectra of PAQS (red) and $PAQS^{2-}$ (green dotted) were calculated by DFT and the calculated difference spectrum in the discharged state (blue) for PAQS in **a** Li system and **b** PAQS in Mg system.

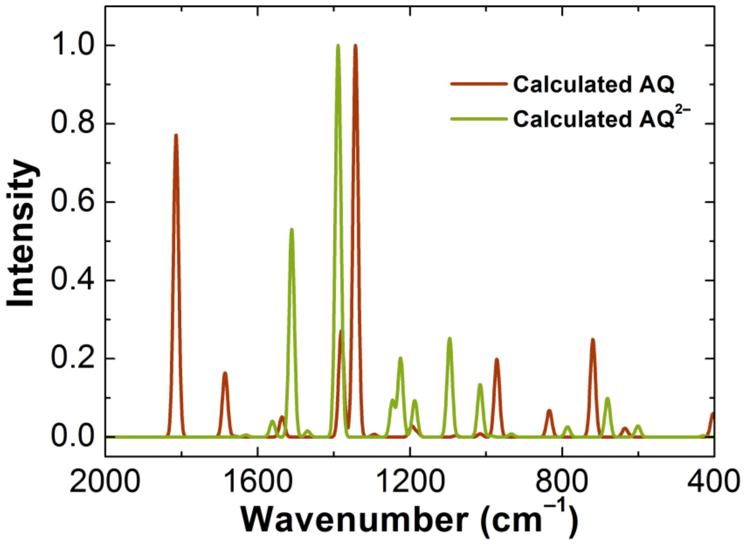

Supplementary Fig. 13 Theoretical spectra of AQ and AQ^{2-}. The theoretical spectra of AQ (red) and AQ^{2-} (green) calculated by DFT.

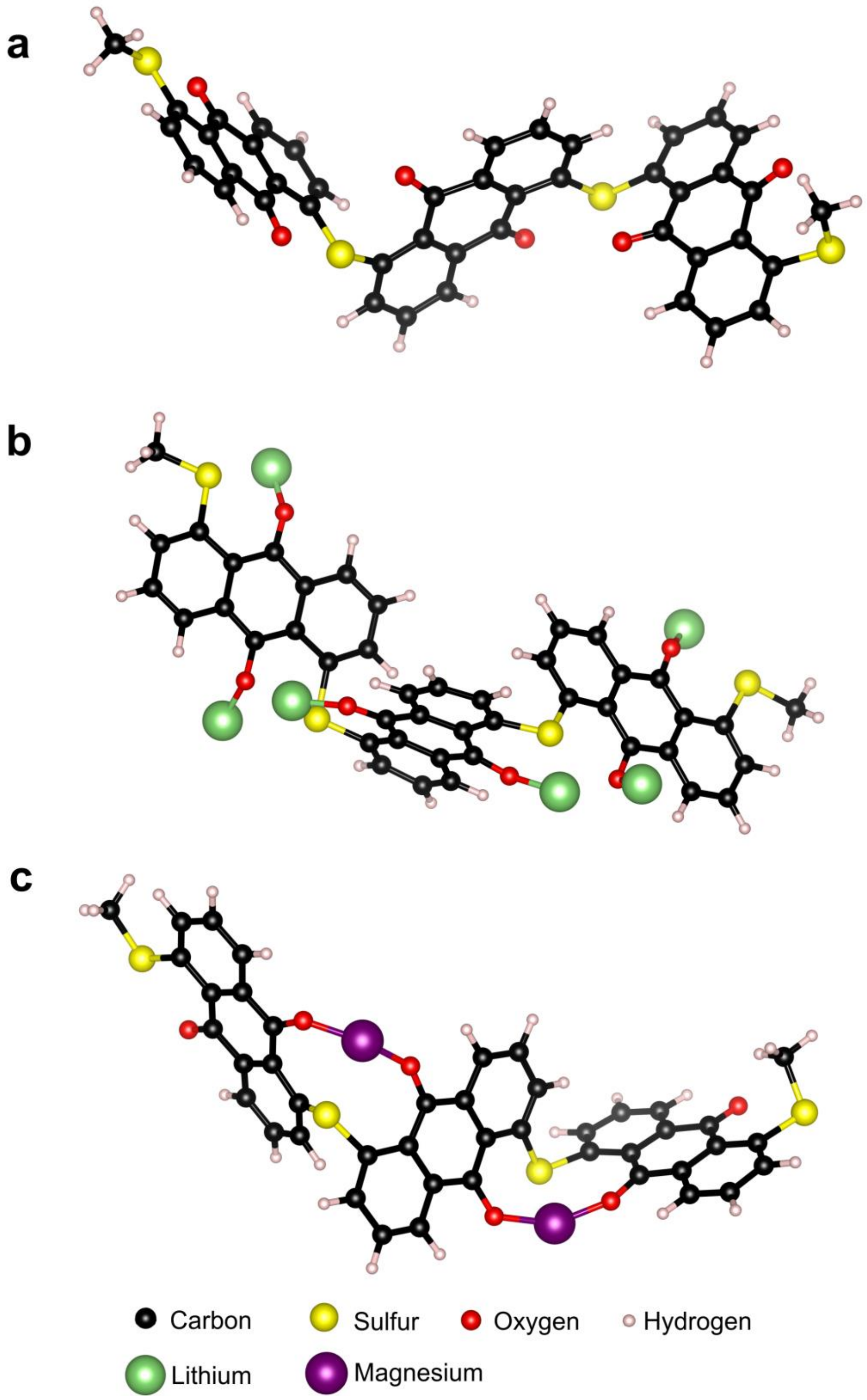

Supplementary Fig. 14 Relaxed geometry of a PAQS, Li-PAQS and Mg-PAQS. Relaxed geometry of a polymeric structure of **a** PAQS, **b** Li-PAQS and **c** Mg-PAQS, modelled with three monomeric units and terminated with methyl group.

Supplementary Table 1. Average bond lengths in charge and discharge point and their relative change in Li and Mg system. Due to the fact that no symmetry was imposed during calculation, the changes of C–C bond lengths on passing from charge to discharge point are different between individual C–C bonds. However, differences in C–C bond lengths are marginal in comparison to the ones observed for C–O bonds.

		**Li system**		**Mg system**	
	**PAQS**	**PAQS^{2-}**	**Change in %**	**PAQS^{2-}**	**Change in %**
**$\langle r_{CO} \rangle$ (Å)**	1.220	1.313	7.6	1.321	8.3
**$\langle r_{CH} \rangle$ (Å)**	1.085	1.085	0	1.085	0
**$\langle r_{CC} \rangle$ (Å)**	1.422	1.417	-0.4	1.419	-0.2

Supplementary Notes 1.

XRD measurements. The powder XRD measurements were carried out on a PANalytical X'pert PRO high-resolution and on a Siemens D5000 diffractometer with Cu Kα_1 radiation (λ = 1.5406 Å). The PAQS was measured in an angle range of 5° to 65° 2θ with the 0.033° step and 100s measuring time per step on zero-background holder (Si wafer) an the AQ in an range 10-60°, step 0.04° in measuring time 1s per step, on zero-background sample holder. The *ex-situ* cathodes were measured inside a Swagelok type cell with a 200 μm thick beryllium window in the the 2θ range from 10° to 40° with the 0.04° step per 100s.
Anthraquinone (AQ) is a crystalline solid that has monoclinic crystal structure, due to the π-π stacking of the anthracene rings (Supplementary Fig. 9, green diffractogram).[1] On the other hand, poly(anthraquinoyl sulfide) (PAQS) exhibits poor crystallinity with low peak intensities as shown in the Supplementary Figure 9 (red diffractogram). These peaks are connected with presence of short chain oligomers of PAQS and not AQ monomers.[1] The PAQS cathodes (fresh (Supplementary Fig. 9, orange diffractogram) and the *ex-situ* cathode in a discharge state (Supplementary Fig. 9, blue diffractogram)) were measured inside a Swagelok type cell with a beryllium window. The fresh cathode resembles the PAQS structure with a slight shift of the peaks due to the height displacement of the thick Be window. The *ex-situ* cathode in the discharge state exhibits a loss in arrangement which is due to formation of a poorly crystalline or amorphous discharged compounds. This emphasises the importance of *in-operando* IR spectroscopy, which is one of the few methods that allow visualization of molecular changes inside PAQS and other organic compounds.

Supplementary Notes 2.

Polyanthraquinone (PAQ) *in-operando* ATR-IR characterization.The PAQ was cycled at 50 mA g^{-1} in the voltage window of 1.5 V to 3.0 V inside the *in-operando* ATR-IR cell. Capacities around 219 mAh g^{-1} were achieved. Similar electrochemical mechanism as in the case of PAQS can be expected for PAQ (Supplementary Fig. 10a). PAQ exhibited high electrochemical activity with a characteristic monotonous change of the voltage between 2.5 and 2.0 V (Supplementary Fig. 10b). Similar changes in the voltage were seen in PAQS. In the IR spectrum (Supplementary Fig. 10c, red curve), PAQ has two characteristic peaks located at 1670 and 1592 cm^{-1}. The two peaks are assigned to the streaching vibration of the C=O and –C=C– bond, respectively.[2] During the discharge, a decrease of intensity for C=O and –C=C– stretching bands and appearance of new $–C–O^{-} Li^{+}$ band (1373 cm^{-1}), can be seen in the differential spectrum. In charge the situation is reversed bands for C=O and –C=C– stretching increase in intensity, while band for $–C–O^{-} Li^{+}$ decreases (Supplementary Fig. 10c).

Supplementary Notes 3.

Polyaniline *in-operando* ATR-IR characterization. Polyaniline (emeraldine base) in Supplementary Figure 11a was cycled at 50 mA g^{-1} in the voltage range from 2.5 (except the first discharge, which was to 2.0 V to achieve lithiation of emeraldine base) to 4.0 V Li/Li^{+} (Supplementary Fig. 11b). Capacities around 137 mAh g^{-1} were achieved. In the IR spectra (Supplementary Fig. 11c, red curve) of the polyaniline (emeraldine base) characteristic peaks are observed. The polyaniline (emeraldine base) has two strong vibrational peaks for the

aromatic ring stretching –C=C– at 1593 and 1502 cm^{-1}. Furthermore, the polyaniline have peaks for –C–H bending and –C–N stretching at 1309, 1167 and 1109 cm^{-1}.[3,4]

During the cycling of polyaniline, changes in the ATR spectra are observed. In the ATR difference spectra (Supplementary Fig. 11c), upon discharge we can see increase of the bands characteristic for EC:DEC (1803, 1776, 1741, 1259, 1159 and 1072 cm^{-1}), consistent with introduction of solvent in polyaniline, and upshift of the bands between 1500 and 1600 cm^{-1}, which is characteristic for changed ratio between phenyl and quinoid rings in favour of phenyl ones. Upon charge the bands connected with the carbonate solvents decrease and the bands in the region between 1500 and 1600 cm^{-1} downshift, which is characteristic for increased amount of quinoid rings (Supplementary Fig. 11c). This observations are in agreement with the recent study made on polyaniline, where cycling behaviour was investigated using electrochemical quartz crystal microbalance,[3] and previous *ex-situ* IR study on model compounds of polyaniline.[4]

Supplementary References

1. Song, Z., Zhan, H. & Zhou, Y. Anthraquinone based polymer as high performance cathode material for rechargeable lithium batteries. *Chem. Commun.* 448–450 (2009).
2. Song, Z. *et al.* Polyanthraquinone as a Reliable Organic Electrode for Stable and Fast Lithium Storage. *Angew. Chem. Int. Ed.* **54,** 13947–13951 (2015).
3. Jiménez, P. *et al.* Lithium n-Doped Polyaniline as a High-Performance Electroactive Material for Rechargeable Batteries. *Angew. Chem. Int. Ed.* **56,** 1553–1556 (2017).
4. Boyer, M.-I. *et al.* Vibrational Analysis of Polyaniline: A Model Compound Approach. *J. Phys. Chem. B* **102,** 7382–7392 (1998).
